# Patient With Post-operative Recurrent Pancreatic Cancer Treated With Cone Beam Computed Tomography-Guided Stereotactic Adaptive Radiotherapy: A Case Report

**DOI:** 10.7759/cureus.75284

**Published:** 2024-12-07

**Authors:** Farnoush Forghani, Alden D'Souza, Eric Laugeman, Allen MO, Pamela P Samson, Hyun Kim

**Affiliations:** 1 Radiation Oncology, Washington University School of Medicine, Saint Louis, USA

**Keywords:** ct-guided adaptive radiotherapy, hypersight, pancreatic cancer, post-operative recurrence, stereotactic radiotherapy (srt)

## Abstract

CT-guided adaptive radiotherapy (ART) for the treatment of pancreatic adenocarcinoma is rapidly increasing and has been shown to provide advanced treatment tools comparable to magnetic resonance imaging (MRI)-guided adaptive therapy. Here, we provide the first case report of a local pancreatic recurrence treatment after definitive resection using cone beam computed tomography (CBCT)-guided ART (CT-guided ART) enabled by HyperSight imaging (Varian Medical Systems, Inc., Palo Alto, CA, USA) for daily delineation of organs-at-risk (OARs) and target to improve the quality of online ART.

A 61-year-old female was diagnosed with pancreatic adenocarcinoma, and her CT demonstrated a 1.9 cm lesion abutting the superior mesenteric vein. She received nine cycles of neoadjuvant mFOLFIRINOX, followed by a Whipple resection, with surgical pathology showing moderate to poorly differentiated adenocarcinoma and negative margins and perineural invasion. Approximately 3.5 months after resection, she was noted on endoscopic ultrasound to have a 1.7 cm lesion near the superior mesenteric artery and was referred to radiation oncology for treatment of local recurrence. She had adaptive stereotactic body radiotherapy to this lesion at 50 Gy in five fractions on the Ethos (Varian Medical Systems, Inc., Palo Alto, CA, USA).

This case report highlights the utility of HyperSight CBCT for OAR and tumor delineation for the treatment of a patient with post-operative recurrent pancreatic cancer using CT-guided ART.

## Introduction

Pancreatic adenocarcinoma is the third leading cause of cancer mortality in the United States, with a five-year relative survival of 12.8% [1-3]. The treatment paradigm of locoregional pancreatic adenocarcinoma involves multiagent chemotherapy with the option of radiation therapy to make resection possible [4-5].

Online stereotactic body radiation therapy (SBRT) adaptive radiotherapy (ART) has been shown to improve treatment outcomes, such as favorable toxicity profiles, and improve overall survival for the treatment of pancreatic malignancies [6-10]. Magnetic resonance imaging (MRI)-guided radiotherapy has shown precise delineation of daily organs-at-risk (OARs) and target volume, which helps to improve the efficiency of the SBRT ART technique [6-8].

Development of ring gantry computed tomography (CT)-based radiotherapy machine with customized advanced software for daily adaptive planning (Ethos, Varian Medical Systems, Inc., Palo Alto, CA, USA)-enabled cone beam computed tomography (CBCT)-guided ART (CT-guided ART) [9-10]. The recent development of a novel on-board imaging platform equipped with rapid-acquisition high-quality CBCT imagers called “HyperSight“ (Varian Medical Systems, Inc., Palo Alto, CA, USA) has further extended the utility of CT-guided ART in pancreatic ablation [11]. Prior to the development of HyperSight, delineation of OARs and targets during the Ethos ART for the pancreas was challenging and required a longer time for the adaptive team to identify the organs. This increased uncertainty in contour delineation and dosimetric outcomes. For non-standard pancreas cases of post-operative recurrence, permanent change in the luminal organs made the contour delineation even more difficult [9,10]. HyperSight technology has significantly improved the quality of organ delineation on daily scans, resulting in improved quality of adaptive planning and delivery [12].

Details of the workflow and dosimetric analysis for CT-guided ART for the pancreas are provided by Kim et al. [9] who report the first case of pancreas treatment using CT-guided ART. At the time of their study, HyperSight technology was not implemented. Here, we provide the first case report of a post-operative recurrence pancreatic cancer treatment that benefits from advanced HyperSight imaging and adaptive technology of the Ethos system.

## Case presentation

A 61-year-old female presented with multiple weeks of flank pain, nausea, and weight loss. Her initial CT demonstrated a 1.9 cm hypoattenuating lesion, with magnetic resonance cholangiopancreatography confirming a 1.9 cm lesion in the pancreatic head abutting the superior mesenteric vein (SMV). A fine-needle aspiration biopsy showed poorly differentiated invasive pancreatic adenocarcinoma, with initial staging at pT1N0M0. She received nine cycles of mFOLFIRINOX with CT imaging demonstrating an unchanged pancreatic head mass with a 180-degree abutment of the SMV. She underwent Whipple resection with negative margins and pathology demonstrating perineural invasion. She received adjuvant gemcitabine/capecitabine for two cycles. Her CT chest/abdomen/pelvis eleven months after diagnosis noted periaortic lymphadenopathy and endoscopic ultrasound noted a 1.7 cm lesion near the superior mesenteric artery.

**Figure 1:**
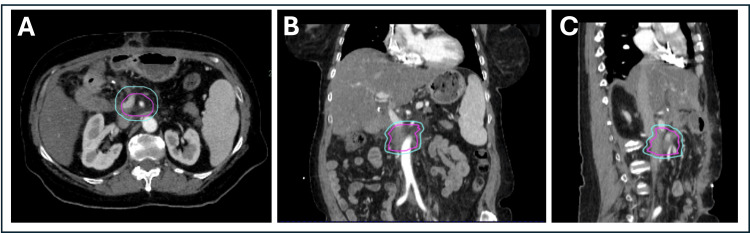
(A-C) Axial, coronal, and sagittal simulation CT images of the patient used for treatment planning. The magenta contour represents GTV/CTV (as they are used interchangeably in our adaptive program); PTV is delineated by cyan contour. CT: computed tomography, GTV/CTV: gross tumor volume/clinical target volume, PTV: planning target volume

She was referred to radiation oncology for treatment of local recurrence. Radiation oncology recommended adaptive SBRT due to the mass’s vicinity to the reconstructed bowel (hepaticojejunostomy and small bowel). Figure 1 shows the disease site at the time of the simulation CT. The patient was treated with 50 Gy in five fractions. She did not have side effects during her radiation treatment or at her two-month follow-up (at the time of this report).

Simulation and contouring

During the time of simulation CT scans, the patient was positioned in a custom immobilization device with the right arm up and left arm at the side to improve beam arrangement for planning, per institutional guidelines for pancreatic adaptive SBRT. Simulated CT with a 2 mm slice thickness was acquired at the end-exhale breath-hold along with a four-dimensional CT to assist in determining the quality of the end-exhale breath-hold. A physicist was present at the time of the simulation CT to review the scans to determine the consistency of the patient’s breathing pattern and the quality of the end-exhale breath-hold scan to use for the initial treatment plan. During the simulation scan, the patient was administered an intravenous contrast (for better delineation of OARs) at the 45-second delay phase per institutional protocol. The patient also received a simulation Ethos scan on the same day, where the patient was positioned on Ethos’ treatment table, and a verification scan was acquired with HyperSight to verify the scan quality in delineation of daily anatomy for online adaptive contouring. In addition, the Identify Surface Monitoring System (Varian Medical Systems, Inc., Palo Alto, CA, USA) was used to test if the patient was compliant with the breath-hold for the duration of treatment. The patient was given breath-hold instructions, and the patient’s ability to breath-hold and the consistency of their breathing pattern for treatment were verified.

The scans were uploaded to Aria (Varian Medical Systems, Inc., Palo Alto, CA, USA), and a fusion review of end-exhale simulation CT with diagnostic CT was performed by a physicist to verify if the scan represents the correct breathing phase and can be used for end-exhale treatment planning. In addition, a diagnostic MRI scan was acquired two months before the CT simulation scan and was fused to the simulation images for assistance in target delineation. The treating radiation oncologist then contoured the clinical target volume (gross tumor volume (GTV)/clinical target volume (CTV)) on the simulation end-exhale scan. Note that CTV and GTV are utilized interchangeably as our institutional practice for adaptive SBRT treatments. A planning target volume (PTV) was generated from a 5 mm uniform expansion of CTV volume (Figure 1).

The critical gastrointestinal post-Wipple luminal organs, stomach, hepaticojejunostomy (HJ), and small and large bowels were contoured at the axial slices from 3 cm below to 3 cm above the PTV location. Because the contrast was only delivered at the time of simulation and not in each treatment session, the density of the contrast was overridden by the density of water. After completion of the contours, a physicist reviewed the contours and the end-exhale scan and made recommendations in planning strategies such as beam geometry and any specific modifications to improve the dosimetric outcome in the plan.

Initial off-line planning

Treatment planning was performed in the Ethos (v.1.1) treatment planning system (TPS). The initial plan that was generated based on the anatomy of simulation CT is called the “reference plan.” A strict isotoxicity approach was used in planning, wherein OAR dose tolerances are prioritized over the target coverage [6,13]. Per our standard ART practices of isotoxicity approach, a PTV optimization structure (PTV_Opt) was generated by subtracting the overlap volume of critical OARs + 5 mm margin from PTV. The PTV_Opt was used in the optimizer to drive the prescription dose to the tumor in areas that have no overlap with OARs [7,14]. Priority 1 and 2 (P1 and P2) objectives and dose constraints for OARs and targets that were used in the optimization are shown in Table 1. A beam geometry of two coplanar beams was used for volumetric modulated arc therapy modality, with 10- and 350-degree collimator angles and 181-80-degree gantry angles avoiding entry through the patient’s left arm. The reference plan was reviewed and evaluated by two physicists and the treating radiation oncologist. The plan was approved for treatment after the patient QA was performed and the Monte-Carlo-based secondary dose calculation (Mobius3D, Varian Medical Systems, Inc., Palo Alto, CA, USA) passed the gamma analysis of 3% dose difference and 2 mm distance-to-agreement criteria.

**Table 1:** P1 and P2 constraints for OARs and target coverage shown for the reference plan, adapted plan (FX#A), and scheduled plan (FX#S) for each fraction with # representing the fraction number. OARs: organs-at-risk, P1 and P2: Priority 1 and 2

Plans	OARs (P1 constraint)					OARs (P2 constraint)		Target coverage (P2 constraint)	
	Stomach	Large bowel	Small bowel	HJ	Spinal cord	Uninvolved liver	Kidneys	PTV	PTV_Opt
	V33 Gy <0.5 cc	V33 Gy <0.5 cc	V33 Gy <0.5 cc	V33 Gy <0.5 cc	D0.5 cc <25 Gy	V20 Gy <33%	Dmean <15 Gy	D95% >50%	D95% >95%
Reference plan	0.01 cc	0.0 cc	0.01 cc	0.04 cc	7.14 Gy	0.0%	9.93 Gy	77.3%	98.7%
FX1 A	0.0 cc	0.0 cc	0.01 cc	0.01 cc	2.40 Gy	0.0 %	1.77 Gy	73.9%	98.1%
FX1 S	0.0 cc	0.0 cc	0.43 cc	3.57 cc	1.43 Gy	0.0 %	2.05 Gy	75.3%	93.4%
FX2 A	0.0 cc	0.0 cc	0.02 cc	0.0 cc	3.09 Gy	0.0 %	2.74 Gy	83.6%	95.3%
FX2 S	0.0 cc	0.0 cc	0.28 cc	1.51 cc	1.54 Gy	0.0 %	2.16 Gy	17.1%	66.4%
FX3 A	0.0 cc	0.0 cc	0.04 cc	0.02 cc	2.41 Gy	0.0 %	2.00 Gy	81.67%	98.8%
FX3 S	0.0 cc	0.0 cc	1.05 cc	0.75 cc	1.50 Gy	0.0 %	2.16 Gy	78.2%	96.4%
FX4 A	0.0 cc	0.0 cc	0.03 cc	0.0 cc	1.55 Gy	0.0 %	2.11 Gy	83.6%	98.1%
FX4 S	0.0 cc	0.0 cc	0.76 cc	0.69 cc	1.91 Gy	0.0 %	2.10 Gy	76.4%	95.3%
FX5 A	0.0 cc	0.0 cc	0.02 cc	0.04 cc	1.94 Gy	0.3 %	2.12 Gy	59.1%	98.3%
FX5 S	0.0 cc	0.0 cc	0.21 cc	9.62 cc	1.98 Gy	0.2 %	2.20 Gy	76.8%	95.8%

Online adaptive treatment planning and delivery

On each treatment session, a high-quality HyperSight CBCT scan of the treatment site was acquired while the patient was on end-exhale breath-hold. After the physicist approved the quality of the scan to use for contouring and planning, Ethos TPS proceeded with performing a deformable registration of the simulation CT to the daily CBCT. Deformable registration was used to account for daily anatomical changes in scans to improve the accuracy of automatic OAR contouring. The OAR contours were automatically propagated from the simulation CT to the daily CBCT and were adjusted using an automatic vendor-supplied artificial intelligence-driven algorithm. The deformable target contours were overridden with rigidly propagated contours to reduce the uncertainty in the target delineation. The OARs that were inside a 3 cm contour ring were adjusted by an advanced practice radiation therapist and reviewed and accepted by the covering radiation oncologist and the physicist to confirm the accuracy. The 3 cm contour ring per standard adaptive protocol is based on the study by Bohoudi et al. [13], who showed limiting the adjustment of the daily OAR contours within the contour ring allows less time for the clinician to re-contour while good OAR sparing and adequate target coverage are maintained. After contouring, Ethos TPS re-optimized the plan based on the anatomy of the day to generate the adaptive plan. The optimization time was ~8.5 minutes. In addition, the reference/simulation-based plan was projected on the anatomy of the day, which is called the “scheduled plan.” Both scheduled and adapted plans were compared by TPS based on P1 and P2 objectives. The covering radiation oncologist and physicist evaluated the adapted and scheduled plans based on isodose volumes and dose-volume histogram (DVH) to decide which plan met the dosimetric goals for OAR sparing and target coverage.

The covering physicist then performed the adaptive plan consistency check and QA using 3D Mobius secondary dose calculation. Upon the acceptance of the adapted plan to use for treatment, the plan was transferred to the treatment machine for delivery. The covering physicist and therapist verified the treatment plan identification, such as plan name and MUs, to ensure the correct plan was used for treatment. A pre-treatment CBCT was acquired to verify that the OARs and target position did not change from the time of the scan, which was used for the adapted plan. More importantly, it was verified in the new scan that no OARs were overlapping with PTV_Opt since this structure was allowed in the plan objective to hold the high isodose regions. The average time between the CBCT used for adaptive planning and pretreatment CBCT for this patient was ~40 minutes. The Identify Surface Monitoring System was used for intrafraction monitoring of the patient’s breathing during the treatment, which assisted the therapists in manually beaming on only when the patient’s breathing was at end-exhale breath-hold.

Dosimetric results

The constraints and target coverage metrics for the reference plan (which was based on simulation CT), adapted plan, and scheduled plan (which were based on daily CBCT) are shown in Table 1. The adapted plan was used for treatment in all five fractions. One can observe that the scheduled plan would have resulted in a violation of the HJ in all five fractions. Additionally, although PTV_Opt coverage was improved by the adaptive plan in FX5, the PTV coverage was significantly reduced by the adaptive plan due to the isotoxicity approach that prioritizes reducing the V33 Gy for HJ from 9.62 cc to 0.04 cc.

**Figure 2:**
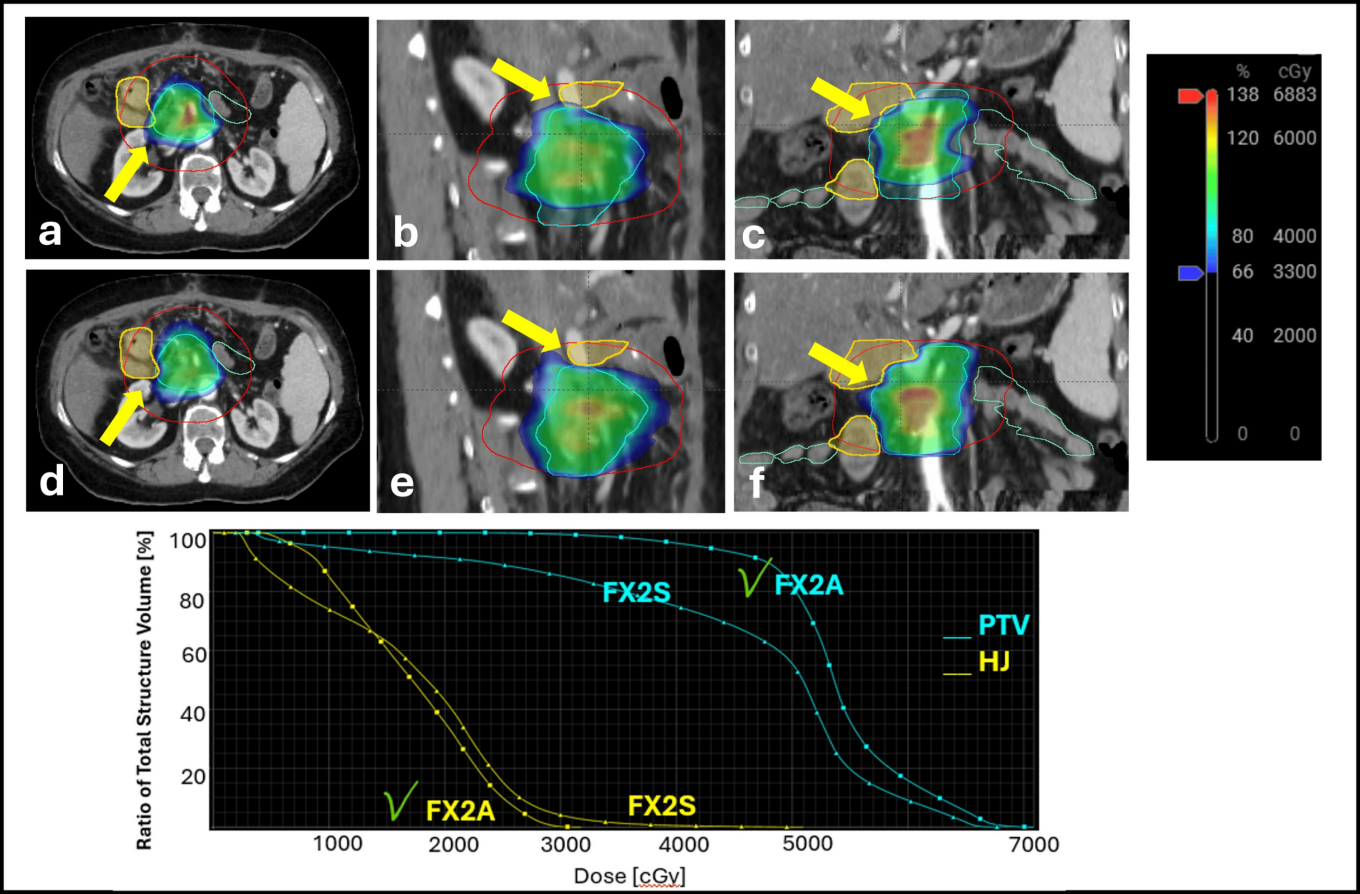
Axial, sagittal, and coronal views of daily CBCT for FX2 of the scheduled plan (top row a-c) and adapted plan (middle row d-f), respectively, with color wash dose overlayed on the scans. Yellow arrows point to the area where the 33 Gy OAR dose tolerance is violated in the scheduled plan for the HJ (yellow contour). On the other hand, in the adapted plan, 33 Gy does not overlap with HJ. Red contour represents the 3 cm contour ring, magenta represents the liver, and orange and green contours represent large and small bowels, respectively. Bottom row is the DVH plot that shows the max dose constraint to HJ was violated in the scheduled plan(FX2S), while this constraint was met in the adapted plan (FX2A). PTV (cyan) is shown in triangle-line for the scheduled plan and square-line for the adapted plan. The green check mark sign in the DVH plot next to FX2A indicates that these constraints were met in the adaptive plan. CBCT: cone beam computed tomography, OAR: organ-at-risk, HJ: hepaticojejunostomy, DVH: dose-volume histogram, PTV: planning target volume

Figure 2 illustrates the dosimetric map and DVH for FX2, where the delivery of the daily adaptive plan has achieved the HJ PI hard constraint, whereas the delivery of the scheduled plan would have violated that constraint.

## Discussion

Here, we present the first reported treatment of a unique case of a post-operative recurrence pancreatic cancer patient using CT-guided ART equipped with newly developed HyperSight CBCT imaging technology. A comparison of the dose constraints between the scheduled plan and the adapted plan showed that treating with the scheduled plan would have resulted in the violation of HJ in all treatment fractions, whereas all the OAR constraints were met when treating with the adapted plan. The high scan quality acquired by HyperSight has increased the quality of OAR and target delineation, resulting in less uncertainty and a higher-quality adaptive plan. In addition, PTV_Opt coverage was improved in the adaptive plan in all five fractions. However, due to the isotoxicity approach that prioritizes the OARs, the PTV coverage was sacrificed in two out of five fractions to reduce the dose to the large volume of HJ.

MR-guided adaptive has proved to improve treatment outcomes such as favorable toxicity profile and overall survival for the treatment of pancreatic malignancies [2,6-8]. Recently, the advancement of Ethos has opened new avenues for the use of CT-guided ART for the treatment of pancreatic cancer. While CT-guided ART has expanded the adaptive pancreatic treatment applications, there are potential limitations compared to MRI-guided ART due to reduced soft tissue contrast and organ delineation [9,10]. The development of HyperSight with advanced image quality has significantly reduced the CT-guided ART limitations in organ delineation [11]. Kim et al. [9] reported the first case of pancreas treatment using CT-guided ART. Our study is novel as it shows that CT-guided ART coupled with extensive quality HyperSight has made it possible to treat a unique and complicated case of organ delineation such as the case reported here. One of the limitations of this study is that it lacks a dosimetric comparison with a similar case treated by an MRI-guided ART modality. However, this study emphasizes the potential of CT-guided ART to be used affordably for the treatment of patients with pancreatic cancer.

## Conclusions

Here, we present the first report of a unique pancreatic post-operative recurrence treated with ART. Due to the difficulty in luminal organ delineation, this case has benefited from the improved image quality of HyperSight technology. As a result, CT-guided ART is an affordable worldwide treatment for pancreatic cancer and may have the potential to provide a comparable treatment quality to MRI-guided ART.
